# Rapid mixing and exchange of deep-ocean waters in an abyssal boundary current

**DOI:** 10.1073/pnas.1904087116

**Published:** 2019-06-18

**Authors:** Alberto C. Naveira Garabato, Eleanor E. Frajka-Williams, Carl P. Spingys, Sonya Legg, Kurt L. Polzin, Alexander Forryan, E. Povl Abrahamsen, Christian E. Buckingham, Stephen M. Griffies, Stephen D. McPhail, Keith W. Nicholls, Leif N. Thomas, Michael P. Meredith

**Affiliations:** ^a^Ocean and Earth Science, National Oceanography Centre, University of Southampton, SO14 3ZH Southampton, United Kingdom;; ^b^National Oceanography Centre, SO14 3ZH Southampton, United Kingdom;; ^c^National Oceanic and Atmospheric Administration Geophysical Fluid Dynamics Laboratory & Atmospheric and Oceanic Sciences Program, Princeton University, Princeton, NJ 08544;; ^d^Department of Physical Oceanography, Woods Hole Oceanographic Institution, Woods Hole, MA 02543;; ^e^Polar Oceans, British Antarctic Survey, CB3 0ET Cambridge, United Kingdom;; ^f^Department of Earth System Science, Stanford University, Stanford, CA 94305

**Keywords:** ocean mixing, overturning circulation, submesoscale instabilities, turbulence

## Abstract

The overturning circulation of the global ocean is critically shaped by deep-ocean mixing, which transforms cold waters sinking at high latitudes into warmer, shallower waters. The effectiveness of mixing in driving this transformation is jointly set by two factors: the intensity of turbulence near topography and the rate at which well-mixed boundary waters are exchanged with the stratified ocean interior. Here, we use innovative observations of a major branch of the overturning circulation—an abyssal boundary current in the Southern Ocean—to identify a previously undocumented mixing mechanism, by which deep-ocean waters are efficiently laundered through intensified near-boundary turbulence and boundary–interior exchange. The linchpin of the mechanism is the generation of submesoscale dynamical instabilities by the flow of deep-ocean waters along a steep topographic boundary. As the conditions conducive to this mode of mixing are common to many abyssal boundary currents, our findings highlight an imperative for its representation in models of oceanic overturning.

## Significance

The overturning circulation of the global ocean is regulated by deep-ocean mixing, which transforms cold waters sinking at high latitudes into warmer, shallower waters. The effectiveness of mixing in driving this transformation is jointly set by the intensity of turbulence near topography and the rate at which well-mixed boundary waters are exchanged with the stratified ocean interior. We use innovative observations of a branch of the overturning circulation in the Southern Ocean to identify a previously undocumented mixing mechanism, by which deep-ocean waters are rapidly laundered through intensified near-boundary turbulence and boundary–interior exchange. As the conditions triggering this mechanism are common to other branches of the overturning circulation, our findings highlight a requirement for its representation in models of the overturning.

Current theories and models of the overturning circulation unequivocally stress the pivotal role of turbulent mixing near topographic boundaries in driving the overturning’s upwelling branch (1, 2). As cold abyssal waters flow above a rough seafloor, bottom-intensified, small-scale turbulence induces mixing with warmer overlying layers, leading to a net warming and upwelling of abyssal waters along topography (3, 4). The intensification of turbulence near topographic boundaries has been extensively demonstrated with observations and attributed to a variety of physical processes (5–7). However, the manner in which topographically localized turbulence gives rise to large-scale, deep-ocean upwelling has been the subject of an enduring debate, focused on the uncertain renewal of stratified waters in the near-boundary mixing zone (8–11). Despite abundant circumstantial evidence of the occurrence of boundary–interior exchange (8, 12–14), the processes determining its rate and interaction with near-boundary turbulence remain undetermined, rendering our understanding of oceanic overturning incomplete.

**Fig. 1.**
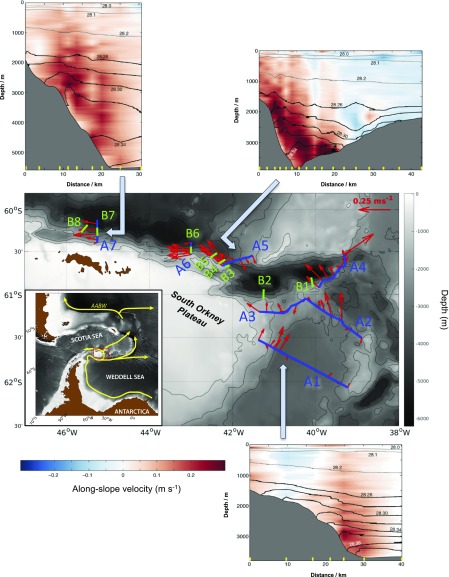
Along-stream evolution of the abyssal boundary current in the Orkney Passage region. The observational domain is shown by the red outline in the (*Inset*) large-area map, with major Antarctic Bottom Water (AABW) pathways indicated in yellow (15, 16). Sections of measurements are marked by blue (coarse-resolution, A1–A7) and green (fine-resolution, B1–B8) lines in the main map. ALR surveys were conducted in the area between sections B3 and B4 (Fig. 3). Observations of horizontal velocity averaged vertically over the AABW layer [defined by neutral density in excess of 28.26 kg m^−3^] are indicated by red vectors, with one vector per station. Bathymetry is denoted by gray shading in both maps. Outer panels show along-slope velocity (shading; positive values indicate flow that is directed equatorward, i.e., with the South Orkney Plateau to its left) and neutral density (contours; darker contours denote isopycnals within AABW) along sections A1, A5, and A7. Yellow ticks at the base of each *Inset* mark the locations of measurement profiles.

To address this important gap, we conducted a set of systematic measurements of the hydrographic, velocity, and shear microstructure properties of a major abyssal boundary current in the Southern Ocean. Abyssal boundary currents convey newly ventilated waters sinking to great depth at high latitudes away from their sources and underpin the deepest limb of the overturning circulation (3, 4). In the boundary current targeted here, Antarctic Bottom Water formed in the Weddell Sea outflows the region by skirting the topographic barrier of the South Scotia Ridge and negotiating the narrow, 3,500-m-deep Orkney Passage (15, 16) (Fig. 1). The observations were obtained in March 20–May 1, 2017 from RRS *James Clark Ross* under the auspices of the UK–US Dynamics of the Orkney Passage Outflow (DynOPO) project (17), and consisted of two core elements: a series of fifteen 6- to 128-km-long vessel-based sections of profile measurements crossing the boundary current at an unusually high horizontal resolution (Fig. 1; 2–10 km for sections A1–A7; ∼350 m for sections B1–B8), and spanning a 350-km-long stretch of the current; and two detailed isobath-following radiator surveys of the boundary flow in the Orkney Passage, performed with the autonomous underwater vehicle Autosub Long Range (ALR; popularly known as Boaty McBoatface) flying at a height of ∼90 m above the ocean floor. Further details of the dataset are given in *Materials and Methods*.

## Evolution of the Abyssal Boundary Current.

An overview of the along-stream evolution of the abyssal boundary current’s structure is provided by Fig. 1. Antarctic Bottom Water, denser than 28.26 kg m^−3^, navigates through and beyond the Orkney Passage by flowing along the steep topographic slope of the Orkney Plateau and undergoes three distinct changes along its path. First, the boundary current deepens by ∼700 m between its entry to and exit from the region (cf. Fig. 1, sections A1 and A7), with approximately half of the descent occurring closely downstream of the main sill at 60.6°S, 42.2°W (cf. Fig. 1, sections A5 and A7). Second, the boundary flow broadens considerably as it traverses the passage. This broadening is manifested in an along-stream flattening of density surfaces over the topographic slope (cf. Fig. 1, sections A1 and A7) and is associated with successive intensification (Fig. 1, between sections A1 and A5) and weakening (Fig. 1, between sections A5 and A7) of the along-slope flow. Third, the boundary current experiences a pronounced lightening as it circulates through the passage, evident in the gradual waning of waters denser than 28.34 kg m^−3^ from the part of the slope shallower than 3,500 m (i.e., the depth of the main sill; cf. Fig. 1, sections A1 and A7). The deepening, broadening, and lightening of the boundary flow suggest that the current undergoes substantial drag, lateral exchange, and turbulent mixing in crossing the passage, respectively. This raises the question as to which mechanism may trigger a concurrent intensification of these seemingly distinct phenomena.

## Mechanism of Mixing and Lateral Exchange.

**Fig. 2.**
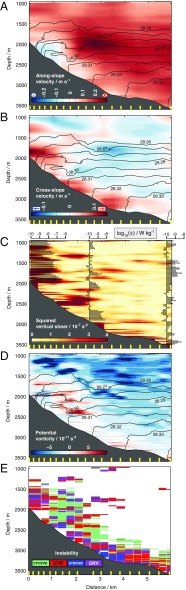
Fine-resolution transect across the abyssal boundary current near the Orkney Passage sill. (*A*) Along-slope velocity (color, with flow direction indicated above the color bar) and neutral density (in kilograms per cubic meter, black contours; only contours within Antarctic Bottom Water are shown) for section B3 (Fig. 1). The mean positions of measurement profiles are marked by yellow tick marks on the lower axis. (*B*) Cross-slope velocity (color) and neutral density (black contours). (*C*) Squared vertical shear (color), neutral density (black contours), and rate of turbulent kinetic energy dissipation (ε, shaded bars). (*D*) Potential vorticity (color) and neutral density (black contours). (*E*) Instability type (CTF = centrifugal, SYM = symmetric, GRV = gravitational, and their hybrids; see *Materials and Methods*).

The governing mechanism is documented by the observations along the high-resolution sections crossing the onshore edge of the boundary current, illustrated here with Fig. 1, transect B3 (Fig. 2). As the boundary current approaches the main sill of the Orkney Passage, it flows along the topographic slope with a peak speed of 0.3 m s^−1^ at ∼800 m above the ocean floor (Fig. 2*A*). Convergence of isobaths along the path of the boundary current predictably initiates the current’s acceleration upstream of the sill (Fig. 1). The high-velocity core of the boundary current is embedded within a cross-slope overturning circulation entailing a downslope near-bottom flow in excess of 0.05 m s^−1^ and a comparable on-slope flow aloft (Fig. 2*B*). The overturning acts to tilt density surfaces near the slope and thereby counteracts, through thermal wind, the intensification of the boundary current on its approach to the main sill (Fig. 1). This results in a reduction of the boundary current’s speed near the ocean floor (Fig. 2*A*).

As the cross-slope circulation extends 2–4 km horizontally away from the topography, it promotes rapid lateral exchange of near-boundary and interior waters. The downslope and on-slope limbs of the overturning are consistently characterized by elevated small-scale turbulence (Fig. 2*C*), with rates of turbulent kinetic energy dissipation (ε ∼ 10^−9^ – 10^−7^ W kg^−1^) and diapycnal mixing (κ ∼ 10^−4^ – 10^−2^ m^2^ s^−1^; *SI Appendix*, Fig. S1*A*) exceeding oceanic background values by typically one to three orders of magnitude. Vigorous turbulence along the overturning’s downslope flow is linked to weak or unstable stratification, manifested as near-vertical isopycnals over a height of 100–500 m above the boundary (Fig. 2 *B* and *C*). The intensification of turbulence within the on-slope flow is, in turn, associated with a series of patches of enhanced vertical shear, with respective cross-slope and vertical scales of 1 km and 100 m. Viewed overall, the cross-slope overturning features pivotally in the local manifestations of all major changes experienced by the boundary current in its journey through the Orkney Passage, thus suggesting that the overturning’s dynamics are key to regulating such changes.

## Dynamics of Mixing and Lateral Exchange.

To elucidate these dynamics, the susceptibility of the boundary flow to submesoscale overturning instabilities is assessed by examining the distribution of potential vorticity (*q*) along transect B3 (Fig. 2*D*). The procedures for this and subsequent calculations are described in *Materials and Methods*. A variety of overturning instabilities may develop in a geophysical fluid when *q* takes the opposite sign to the planetary vorticity (18, 19), which is negative in the Southern Hemisphere. These instabilities induce an overturning circulation that extracts energy from the background flow and expends it in the production of small-scale turbulence, mixing the fluid toward a state of marginal stability. The bulk of the transect is characterized by negative values of *q* on the order of −5 × 10^−11^ s^−3^, indicative of stable conditions. However, substantial patches of positive *q* approaching 5 × 10^−11^ s^−3^ occur systematically within a broad swath extending over respective horizontal and vertical distances of 1–2 km and ∼500 m away from the topographic slope. The fulfillment of the instability criterion in this near-boundary zone indicates that the cross-slope overturning (Fig. 2 *A* and *B*) and the along-stream changes in the boundary current that the overturning brings about (Fig. 1) are associated with instability of the boundary flow.

Overturning instabilities are termed gravitational, symmetric, or centrifugal if the fluid’s vertical stratification, horizontal stratification, or relative vorticity, respectively, is responsible for meeting the instability criterion, in which case instabilities extract energy from the available potential energy, vertical shear, or lateral shear of the background flow (19–21). The nature of the instability experienced by the boundary current is assessed by quantifying the relative importance of the three above factors contributing to the instability criterion in the B3 transect data (*Materials and Methods*). The boundary current is revealed to be primarily subject to centrifugal and symmetric instabilities (Fig. 2*E*) triggered by the large anticyclonic relative vorticity (*SI Appendix*, Fig. S1*A*) and vertical shear/horizontal stratification (*SI Appendix*, Fig. S1 *B* and *C*), respectively, that characterize the current’s onshore edge. Gravitational instability is common only within the ∼100-m-thick layer adjacent to the topographic slope, in which the downslope flow injects relatively light water beneath denser water (Fig. 2*B*). Centrifugal and symmetric instabilities are found to extract energy from the lateral and vertical shears of the background flow at rates of 10–100 mW m^−2^ that are broadly in balance with the measured rates of turbulent kinetic energy dissipation (*SI Appendix*, Fig. S2*A*). Energy supply linked to gravitational instability is generally modest. This basic consistency between sources and sinks of turbulent kinetic energy substantiates the involvement of the cross-slope overturning in generating the elevated small-scale turbulence observed within the boundary current (Fig. 2*C*).

## Submesoscale Instabilities Along Topography.

**Fig. 3.**
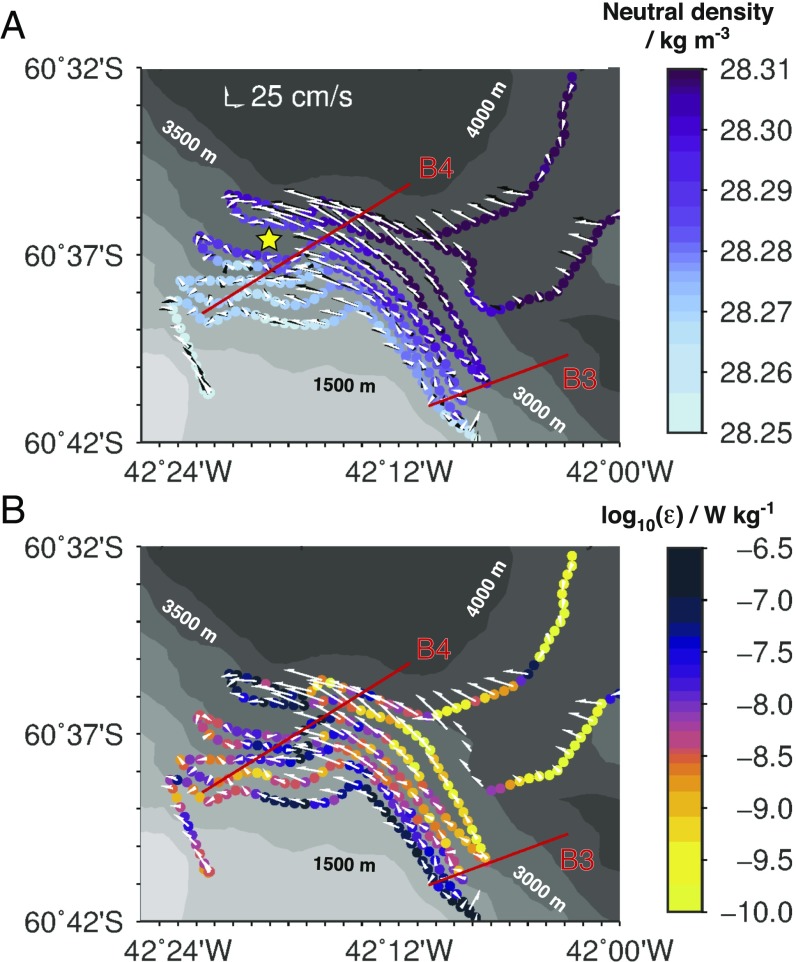
ALR survey of the abyssal boundary current at the Orkney Passage sill. (*A*) Neutral density at ∼90 m above the ocean floor (color), and horizontal velocity averaged over 50–75 m (black vectors) and 125–150 m (white vectors) above the ocean floor. The approximate center of the anticyclonic vortex downstream of the sill is indicated by a yellow star. (*B*) Rate of turbulent kinetic energy dissipation at ∼90 m above the ocean floor (ε, color), and horizontal velocity averaged over 125–150 m above the ocean floor. In both panels, bathymetry is denoted by gray shading, and transects B3 and B4 are indicated by red lines.

While our preceding characterization of the mechanism of boundary current instability is founded on a single high-resolution transect, its generality and underpinning of major changes along the entire stretch of the current spanned by our observations may be illustrated in two ways. First, a systematic reversal in the sign of *q* near topography is common to all high-resolution sections, as is the correspondence of that reversal with conditions favorable to the onset of centrifugal and symmetric instabilities and with elevated turbulence (*SI Appendix*, Fig. S3). These associations occur both in areas of boundary current acceleration along a steepening topographic slope with converging isobaths (e.g., transect B3 in Fig. 2), and in areas of boundary current deceleration along a flattening topographic slope with diverging isobaths (e.g., transect B4 in *SI Appendix*, Fig. S3). Second, evidence of the instabilities’ development following the boundary current past the main sill of the Orkney Passage is provided by the ALR near-bottom observations (Fig. 3). These reveal a widespread downslope flow of relatively light waters at a rate of 0.02–0.05 m s^−1^ that generally intensifies with depth (Fig. 3*A*), consistent with the embedding of ALR within the lower limb of the cross-slope overturning measured in transect B3 (Fig. 2 *A* and *B*) and with the overturning’s implication in deepening and broadening the boundary current. Active growth of instabilities along the boundary flow is suggested by the occurrence of a pronounced anticyclonic vortex with a diameter of ∼5 km as the current passes the main sill (Fig. 3*A*). The vortex resembles anticyclonic structures emerging in numerical simulations of centrifugal instability of flow past topography (22, 23), in that it exhibits a radial shear (of up to ∼40 cm s^−1^ in 2–3 km) that is sufficiently large to produce positive *q*. Generation of the vortex may implicate the tilting of the onshore edge of the boundary current by the cross-slope circulation (*SI Appendix*, Supplementary Text), a mechanism analogous to that forming tornadoes in the atmosphere (24) and intrathermocline eddies in upper-ocean fronts (25, 26). As for transect B3, turbulent kinetic energy dissipation is systematically enhanced along the boundary current’s edge in the ALR measurements (Fig. 3*B*). These feature a colocation of the most intense rates of downslope flow and dissipation around the anticyclonic vortex, and thereby reaffirm the association of cross-slope overturning with small-scale turbulence along the boundary current.

**Fig. 4.**
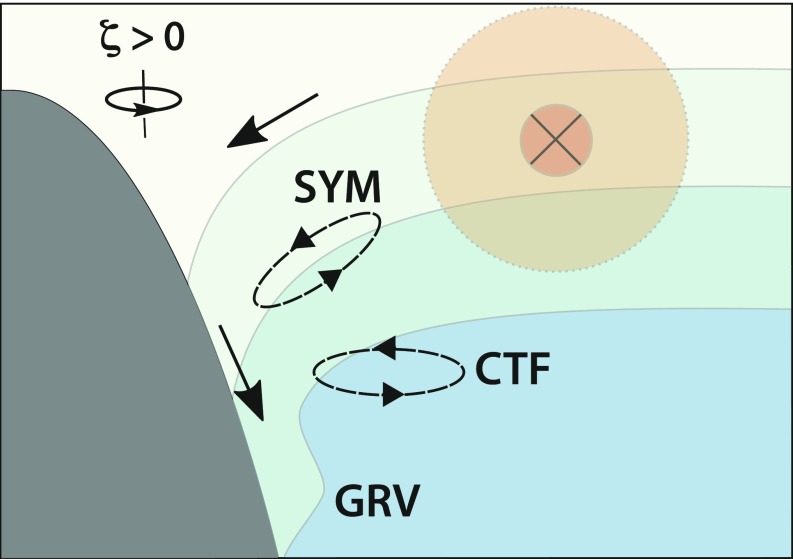
Schematic of the mechanism of mixing and exchange in an abyssal boundary current. The direction of the boundary current is indicated by the cross and the direction of the cross-slope overturning is denoted by solid black arrows. Surfaces of constant density are shown as interfaces between blue-shaded layers. The sense of rotation of the boundary current’s onshore edge is indicated in the upper axis (ζ = relative vorticity). Topographic stress on the boundary current induces a downslope flow, which advects light water under dense and thereby promotes gravitational (GRV) instability and vigorous mixing near the boundary. Centrifugal (CTF) and symmetric (SYM) instabilities occur further from the boundary and drive a lateral exchange of well-mixed near-boundary waters and stratified interior waters.

In conclusion, our targeted measurements of the abyssal boundary current conveying Antarctic Bottom Water through the Orkney Passage show that centrifugal and symmetric instabilities above the topographic slope underpin the intensified drag, lateral exchange, and turbulent mixing experienced by the boundary flow in navigating the passage. A high-resolution numerical model of the region (27), which successfully reproduces all of the basic dynamical features of the observations (*SI Appendix*, Supplementary Text and Figs. S6–S8), indicates that the mechanism is triggered by a topographic frictional stress (28–30) acting on the boundary current’s onshore edge. The stress induces a flow to the right of the current that advects relatively light water down the slope, tilting isopycnals toward the vertical and compressing them horizontally (Fig. 4). This flow leads to a progressive reduction of vertical stratification and enhancement of lateral stratification and shear to the point that these lateral gradients become large enough for the flow to develop centrifugal and symmetric instabilities. The instabilities promote a cross-slope overturning circulation that conveys well-mixed near-boundary waters away from the slope and replenishes the near-boundary zone with more strongly stratified offshore waters. Waters implicated in the boundary–interior exchange are subject to vigorous turbulent mixing, sustained by the overturning’s extraction of energy from the boundary current.

The mechanism of deep-ocean mixing highlighted here is remarkable in that it entails a concurrent intensification of boundary–interior exchange and turbulent mixing, and thereby enables topographically localized turbulence to communicate its effects over potentially much larger scales than that of the near-boundary mixing zone. As the mechanism is activated by a topographic stress-induced downslope flow, it is likely to be of widespread relevance to abyssal boundary currents elsewhere. Abyssal boundary currents commonly flow in the direction of Kelvin wave propagation, i.e., they are generally oriented with the coastline to their left (right) in the Southern (Northern) Hemisphere (3, 4). This is precisely the direction of flow required for the onset of the mixing mechanism documented here, which is predicted to occur over a wide range of topographic slopes and stratification conditions by linear instability theory (*SI Appendix*, Supplementary Text). As explicit resolution of the mechanism (with respective horizontal and vertical scales of hundreds and tens of meters) will long be beyond the capability of even the most sophisticated models of the global ocean circulation, the development of a parameterization of submesoscale dynamical instabilities of abyssal boundary currents stands out as a future priority.

## Materials and Methods

### DynOPO Dataset.

A set of targeted measurements of the hydrographic, velocity, and shear microstructure properties of the abyssal boundary current flowing through the Orkney Passage region, between the Weddell and Scotia Seas in the Southern Ocean, was collected during expedition JR16-005 of RRS *James Clark Ross* between March 20 and May 1, 2017, supported by the UK–US Dynamics of the Orkney Passage Outflow project (Fig. 1). The measurement program consisted of two core elements. In the first, 15 sections of profile observations were conducted across the boundary current, oriented normally to the local topographic slope. Seven of these transects (Fig. 1, A1–A7) extended over a distance of 17–128 km with 7–17 discrete stations at a horizontal spacing of 2–10 km and had the purpose of documenting along-stream changes in the structure and properties of a ∼350-km-long segment of the boundary current. At each station, vertical profiles of hydrographic variables and [horizontal and vertical (31)] velocity were acquired with a conductivity–temperature–depth/300-kHz lowered acoustic Doppler current profiler (CTD/LADCP) package lowered from the vessel, and a free-fall Rockland Scientific VMP-6000 profiler equipped with shear microstructure sensors was deployed concurrently to measure the rate of dissipation of turbulent kinetic energy (ε) down to ∼50 m above the ocean floor. The remaining eight sections (Fig. 1, B1–B8) involved towing the CTD/LADCP package in a seesaw pattern through the water column deeper than 1,000 m with the vessel steaming at ∼1 kn over a distance of 6–12 km, yielding a characteristic horizontal resolution of measurements of ∼350 m. These transects sought to enable a detailed assessment of the dynamics of the boundary current’s interaction with the sloping topography of the Orkney Passage. Subsequent to some high-resolution sections (Fig. 1, B3, B4, and B6), vertical profiles of microstructure measurements were obtained at several points along the section with the VMP-6000 profiler. Full details of the dataset acquisition may be found in the JR16-005 cruise report (32).

The second element of the measurement program entailed the performance of two focused surveys of the boundary current around the main sill of the Orkney Passage with the autonomous underwater vehicle Autosub Long Range (ALR; popularly known as Boaty McBoatface). One of these surveys (termed M44) is considered here. In its M44 mission, ALR traveled 178 km in 75.4 h at a mean height of 91 m (with a SD of 10 m, and a range of 60–140 m) above the ocean floor, following a radiator track on the western slope of the passage with individual legs extending 17–20 km along an isobath, and successive legs separated by a depth of 250 m (Fig. 3 and *SI Appendix*, Movie S1). The mission concluded with a pair of transects aligned parallel to the sill. ALR was equipped with a CTD, 300-kHz upward- and downward-looking ADCPs, and a Rockland Scientific MicroRider microstructure module to obtain measurements of hydrographic variables, horizontal velocity (averaged over a vertical distance of 50 m) above and below the vehicle, and ε, respectively. The processing procedure for all variables is described in ref. 32.

### Calculation of Turbulent Dissipation and Mixing Rates from Microstructure Measurements.

The rate of dissipation of turbulent kinetic energy, ε, was computed from VMP-6000 and ALR microstructure measurements as $\varepsilon =7.5\nu \bar{{\left(\partial u'/\partial l\right)}^{2}}$, where ν is the molecular viscosity and $\bar{{\left(\partial u'/\partial l\right)}^{2}}$ is the variance in the shear in the direction of sampling (denoted by l; vertical for the VMP-6000, and quasi-horizontal for the ALR) of the velocity on the plane normal to sampling, over the resolved turbulent wavenumber range (33). The microstructure sampling rate of both instruments was 512 Hz. Shear variance was calculated from VMP-6000 (ALR) measurements every 0.5 m (2 m), using shear spectra computed over a bin width of 1 s (4 s) and integrated between 1 Hz (0.5 Hz) and the spectral minimum in the 10–25 Hz (4–25 Hz) band, or the 25–100 Hz band for ε > 10^−7^ W kg^−1^. The noise floor in VMP-6000 (ALR) ε data was assessed as lower than 10^−10^ W kg^−1^ (10^−9^ W kg^−1^) (32). The rate of turbulent diapycnal mixing, κ, was estimated from VMP-6000-measured ε as $\kappa =\Gamma \varepsilon /N^{2}$, where Γ is a mixing efficiency (taken as 0.2 as pertinent to shear-driven turbulence) and *N* is the buoyancy frequency (34).

### Calculation of Potential Vorticity.

The Ertel potential vorticity, *q*, is defined as $q=\left(f\hat{k}+\nabla \times u\right)\cdot \nabla b$, where f is the Coriolis parameter, $\hat{k}$ is the vertical unit vector, **u** is the 3D velocity vector, and $b=-g\rho /\rho_{0}$ is the buoyancy (g is the acceleration due to gravity, ρ is density, and $\rho_{0}$ is a reference density) (18). To calculate *q* along the high-resolution transects in Fig. 1, B1–B8 (Fig. 2*D* and *SI Appendix*, Fig. S3), we adopted the approximation $q\approx \left(f+\partial v/\partial x\right)N^{2}-\left(\partial v/\partial z\right)\left(\partial b/\partial x\right)$, where (u,v) is the horizontal velocity vector referenced to the cross-slope (*u*) and along-slope (*v*) directions; *x, y* and *z* refer to the cross-slope, along-slope, and vertical distances, respectively; and *N* is the buoyancy frequency. This approximation is associated with two possible sources of error. First, the vertical component of relative vorticity, ζ=∂v/∂x−∂u/∂y, where *y* denotes the along-slope distance, is approximated by its first term, i.e., ζ≈∂v/∂x. Although this may underestimate the magnitude of ζ by up to a factor of 2 in the limit of the flow adopting solid body rotation (35), the boundary current’s close alignment with isobaths suggests that this limit is seldom approached. Despite this possible bias, ζ regularly exceeds *f* by as much as a factor of 2 in areas of the high-resolution transects where *q* is positive (*SI Appendix*, Fig. S1*A*). Our diagnostics of overturning instabilities may thus be viewed as quantitatively conservative, and qualitatively robust to this source of error. Second, it is assumed that |(∂v/∂z)(∂b/∂x)|≫|(∂u/∂z)(∂b/∂y)|. This approximation likely leads to negligible error, as geostrophy dictates that horizontal buoyancy gradients associated with the boundary current must be aligned perpendicular to the (predominantly along-slope) orientation of the flow (Fig. 2 *A* and *B*), such that |(∂b/∂x)|≫|(∂b/∂y)|. The high-resolution model diagnostics of *q* presented further below confirm that both of these approximations do not affect the robustness of our results.

### Characterization of Overturning Instabilities.

Overturning instabilities develop in areas where fq<0 (18, 19). This criterion may be equivalently expressed as $\phi_{Ri_{B}}<\phi_{c}$ (20, 21), where the balanced Richardson number angle $\phi_{Ri_{B}}={\text{tan}}^{-1}\left(-N^{-2}{\left|\partial v/\partial z\right|}^{2}\right)$ and the critical angle $\phi_{c}={\text{tan}}^{-1}\left(-1-f^{-1}\nabla \times u\cdot \hat{k}\right)\approx {\text{tan}}^{-1}\left(-1-f^{-1}\left(\partial v/\partial x\right)\right)$. The same approximations as in the calculation of *q* were adopted here, with a further two assumptions. First, the flow is assumed to be significantly influenced by geostrophic dynamics. This is supported by the broad agreement between the measured vertical shear in the along-slope flow and the geostrophic shear on horizontal scales of O(1 km) (*SI Appendix*, Fig. S1 *B* and *C*). Second, the instabilities’ basic properties are assumed to be unaffected by 3D effects (36) or the presence of a solid boundary (37). The validity of this assumption is endorsed by the good agreement between our observation-based characterization of instabilities and the results of a high-resolution model capturing the full dynamics, discussed below. When the instability criterion is met, the nature of the instability may be determined from the value of $\phi_{Ri_{B}}$ (20, 21) (Fig. 2*E* and *SI Appendix*, Fig. S3). Gravitational instability is associated with $-180{}^{\circ}<\phi_{Ri_{B}}<-135{}^{\circ}$ and *N*^*2*^ < 0. Gravitational–symmetric instability corresponds to $-135{}^{\circ}<\phi_{Ri_{B}}<-90{}^{\circ}$ and *N*^*2*^ < 0. Symmetric instability is indicated by $-90{}^{\circ}<\phi_{Ri_{B}}<-45{}^{\circ}$, with *N*^*2*^ > 0 and $f^{-1}\nabla \times u\cdot \hat{k}$ ≤ 0; or by $-90{}^{\circ}<\phi_{Ri_{B}}<\phi_{c}$, with *N*^*2*^ > 0 and $f^{-1}\nabla \times u\cdot \hat{k}$ > 0. Symmetric–centrifugal instability is implied by $-45{}^{\circ}<\phi_{Ri_{B}}<\phi_{c}$, with *N*^*2*^ > 0 and $f^{-1}\nabla \times u\cdot \hat{k}$ < 0.

This set of instability diagnostics may be qualitatively validated by comparison with the outcome of a different approach, grounded on the same fundamental dynamics yet free of the assumption of geostrophic balance and of the explicit computation of derivatives. In this alternative framework, the instability properties of the flow are expressed in the distribution of absolute momentum, defined as M=v+fx (38). Conditions favorable to centrifugal instability are manifested in a reversal in the cross-slope gradient of M. Susceptibility to symmetric instability is highlighted by the steepness of isopycnals exceeding that of M surfaces, in the presence of gravitationally stable stratification. Examination of the structure of M for transect B3 (*SI Appendix*, Fig. S1*D*) reveals a general association of the centrifugally and symmetrically unstable areas identified by this approach with those diagnosed from the balanced Richardson number (Fig. 2*E*). For example, the green-shaded area in the 1–3-km distance range in Fig. 2*E*, indicative of conditions favorable to symmetric–centrifugal instability, corresponds to a prominent reversal in the cross-slope gradient of M in *SI Appendix*, Fig. S1*D*. Similarly, the red- and blue-shaded band extending along the deepest ∼300 m in Fig. 2*E*, which denotes susceptibility to symmetric instability, is characterized by M surfaces that are regularly flatter than isopycnals in *SI Appendix*, Fig. S1*D*.

The energy sources associated with the overturning instabilities are assessed in *SI Appendix*, Supplementary Text.

### Data Availability.

Data deposition: All observational data sets obtained during the JR16-005 expedition have been deposited at the British Oceanographic Data Centre (https://www.bodc.ac.uk/resources/inventories/cruise_inventory/report/16299/) (17). Numerical model data are available via Zenodo (doi:10.5281/zenodo.3240623) (27).

## Supplementary Material

Supplementary File

Supplementary File
