# Supplementary material for: Rapid mixing and exchange of deep-ocean waters in an abyssal boundary current
Source: Proc Natl Acad Sci U S A. 2019 Jun 18;116(27):13233–8. doi: 10.1073/pnas.1904087116 (PMC6613131; doi:10.1073/pnas.1904087116)
Supplement: Supplementary File [file pnas.1904087116.sapp.pdf]

Supporting Information

Rapid mixing and exchange of deep-ocean waters in an abyssal boundary current

Supplementary Text

Characterization of energy sources associated with overturning instabilities.

Overturning instabilities derive their kinetic energy from a combination of convective available potential energy (gravitational instability), vertical shear production (symmetric instability) and lateral shear production (centrifugal instability) **(1)**. The characteristic rates of energy extraction from these three sources were estimated from CTD / LADCP measurements of buoyancy and (horizontal and vertical) velocity along transect B3, to assess whether the observed dissipation can be plausibly sustained by the instabilities. The approach detailed in **(2)** was followed. The rate of extraction of available potential energy was estimated as $F_b = \overline{w'b'}$, where w is vertical velocity, the overline denotes a spatial average over the area of the instability, and primes indicate the deviation from that average. The spatial average was computed horizontally at each depth level along the entire transect, to capture the buoyancy flux induced by the vertical flows occurring within the area of the instabilities (Fig. 2e). The rates of vertical and lateral shear production were estimated as $P_{vrt} = -\overline{\boldsymbol{u_h}'w'} \cdot (\partial\overline{\boldsymbol{u_h}}/\partial z)$ and $P_{lat} = -\overline{\boldsymbol{u_h}'v_s'} \cdot (\partial\overline{\boldsymbol{u_h}}/\partial s)$, respectively, where $\boldsymbol{u_h}$ is the horizontal velocity vector, s is the horizontal coordinate perpendicular to the depth-integrated flow, and v_s is the component of $\boldsymbol{u_h}$ in that direction. Here, the spatial average was calculated vertically at each horizontal location, to determine the momentum fluxes associated with the cross-slope overturning flow (Fig. 2b).

The results of this calculation are summarized in *SI Appendix*, Fig. S2a, which provides an overview of the transect- and depth-integrated values of F_b, P_{vrt} and P_{lat}, and compares them with the range of the depth-integrated rate of turbulent kinetic energy

dissipation measured at the three microstructure stations on the section (Fig. 2c). The comparison suggests that the observed dissipation may be plausibly sustained by the overturning instabilities, and that centrifugal and symmetric instabilities dominate the supply of energy to small-scale turbulence. The latter inference is consistent with the prevalence of those instabilities in the instability diagnostics in Fig. 2e. The relative unimportance of F_b also indicates that other instabilities associated with a release of available potential energy, such as baroclinic instability, are unlikely to play a significant role in the near-boundary dynamics of the boundary current. This result is in line with the predictions of linear instability theory **(3)**, according to which a near-boundary flow characterized by a slope Burger number $S = (f^{-1}N \tan\theta)^2 \gg 1$ (where θ is the topographic slope), such as the boundary current in the Orkney Passage, is more susceptible to centrifugal and symmetric instabilities than to baroclinic instability over a wide range of balanced Richardson numbers.

This indicative demonstration of a broad energetic consistency between the sources and sinks of turbulent kinetic energy at the abyssal boundary current's edge is founded on a single transect (B3), for which the boundary flow distinctively accelerates as the topographic slope steepens and isobaths converge toward the Orkney Passage sill (Fig. 1). The demonstration's wider relevance may be illustrated by repeating the analysis in a contrasting transect (B4), for which the boundary current decelerates as the topographic slope relaxes and isobaths diverge downstream of the sill (Fig. 3). Again, it is found (*SI Appendix*, Fig. S2b) that the observed dissipation (*SI Appendix*, Fig. S3) may be plausibly sustained by the overturning instabilities, with a prevalence of centrifugal and symmetric instabilities in transferring energy to small-scale turbulence.

Generation of the anticyclonic vortex downstream of the Orkney Passage sill.

The development of centrifugal and symmetric instabilities along the boundary current is illustrated most clearly by the ALR measurements straddling the Orkney Passage sill, and the high-resolution sections B3-B4 bounding the area of the ALR survey (Fig. 3). Immediately downstream of the sill, an abrupt reduction in the topographic slope occurs, apparent in the divergence of the 1500 m and 3500 m isobaths near 60° 38'S, 42° 12'W (Fig. 3). The along-slope boundary flow (*SI Appendix*, Fig. S4a) is expected to broaden and decelerate in response, resulting in a relaxation of the topographic stress and associated downslope flow that set up the large lateral stratification and shear (*SI Appendix*, Fig. S1a) triggering the condition for instability, $fq < 0$, at transect B3 upstream of the sill (Figs. 2d,e). This expectation is confirmed by the observed cross-slope flow along section B4 (*SI Appendix*, Fig. S4b), which exhibits a reversal in the sense of cross-slope overturning with respect to transect B3. At B4, upslope near-bottom flow in excess of 0.1 m s^{-1} (*SI Appendix*, Figs. S4b-c), and comparable off-slope flow aloft (*SI Appendix*, Fig. S4b), drive a flattening of isopycnals and a lateral expansion of the boundary current's onshore edge (*SI Appendix*, Fig. S4a), and thus act to suppress the condition for instability (*SI Appendix*, Fig. S3). This is consistent with an active growth of the instabilities occurring as the boundary current flows past the sill.

A plausible result of the instabilities' development is the generation of the intense anticyclonic vortex observed by ALR downstream of the sill. To elucidate the generation mechanism, we express the Ertel potential vorticity as $q = q_v + q_h$, where $q_v = (f + \zeta)N^2$ is the vertical contribution to q associated with the vertical components of the absolute vorticity, $(f + \zeta)$, and the stratification, N^2; and $q_h = \boldsymbol{\omega_h} \cdot \boldsymbol{\nabla_h} b$ is the baroclinic contribution to q associated with the horizontal components of the absolute

vorticity, $\boldsymbol{\omega_h}$, and the stratification, $\boldsymbol{\nabla_h} b$ **(4)**. As the cross-slope overturning measured at transect B4 (*SI Appendix*, Figs. S4b-c) acts to reduce both the cross-slope buoyancy gradient and the vertical shear in the along-slope flow, it leads to a decrease in the magnitude of q_h (i.e. q_h moves toward zero). The occurrence of these reductions around the sill may be appreciated by contrasting the distributions of density and q_h at section B3 (*SI Appendix*, Fig. S5a) with those at transect B4 (*SI Appendix*, Fig. S5b). Conservation of potential vorticity then implies that q_v must also move toward zero or, equivalently, that anticyclonic vorticity must increase to move $(f + \zeta)$ toward zero. This is consistent with the measured general increase in the area of near-zero q_v between sections B3 (*SI Appendix*, Fig. S5a) and B4 (*SI Appendix*, Fig. S5b). In summary, the cross-slope overturning downstream of the sill converts vertically sheared flow into laterally sheared flow with anticyclonic vorticity, and may thus conceivably produce the anticyclonic vortex documented by ALR.

To substantiate this mechanism's involvement in vortex generation, the amplitude of the conversion of vertically sheared flow into laterally sheared flow, $\Delta\zeta$, may be estimated as $\Delta\zeta \sim -(\partial v/\partial z) \times (\partial w/\partial x) \times \Delta t$, where $(\partial v/\partial z)$ is the vertical shear of the along-slope flow, $(\partial w/\partial x)$ is the cross-slope gradient of the vertical flow, and Δt is the time over which the cross-slope overturning acts to tilt the vertically sheared flow. Changes in vertical relative vorticity due to meridional advection of planetary vorticity, stretching of vertical vorticity, and frictional torques **(5)** are considered negligible. Adopting characteristic values of $(\partial v/\partial z) \sim 2 \times 10^{-4}$ s^{-1} (Fig. 2a and *SI Appendix* , Fig. S4a), $(\partial w/\partial x) \sim -3 \times 10^{-5}$ s^{-1} (*SI Appendix*, Fig. S4c), and $\Delta t \sim 5 \times 10^{4}$ s (the approximate time elapsed in travelling half the distance between transects B3 and B4 at 0.1 m s^{-1}), we obtain $\Delta\zeta \sim 3 \times 10^{-4}$ s^{-1}. The magnitude of this anticyclonic vorticity is comparable to that of $f \sim -1.3 \times 10^{-4}$ s^{-1}, and thereby suggests that the observed

vortex may be realistically generated via tilting of the along-slope boundary flow by the cross-slope overturning.

Although the generation of a 5 km-wide vortex by a cross-slope overturning with a substantially smaller (~1 km) vertical scale may appear counter-intuitive, its plausibility is illustrated by observations and models of the analogous vortex generation mechanism in the upper ocean. Vortex tilting by secondary circulations at upper-ocean fronts (e.g., in the formation of intra-thermocline eddies **(4)** and tropical instability vortices **(5)**) has been shown to generate anticyclones that are considerably wider than their originating front or secondary circulation. The reason for this is that vortex generation is an intrinsically three-dimensional process: vortex tilting creates a strip of anticyclonic vorticity which, when moving downstream with the frontal flow, can wrap into a coherent vortex that may span many times the width of the strip. While in the upper ocean the wrapping of the vortex strip is typically triggered by frontal meanders, in our abyssal boundary current example it is likely elicited by along-stream variations in topographic slope and flow separation – such as are seen in the current's transit across the sill, between sections B3 and B4 (Fig. 3).

Numerical simulation of the Antarctic Bottom Water flow through the Orkney Passage.

A hierarchy of numerical simulations of the Antarctic Bottom Water flow through the Orkney Passage was conducted to corroborate our interpretation of the measurements, and gain further insight into the dynamics of the abyssal boundary current.

First, a low-resolution (1 km in the horizontal, 63 m in the vertical) hydrostatic simulation was performed with the Massachusetts Institute of Technology general

circulation model (MITgcm) **(6)** in a domain extending to the north and east of the Orkney Plateau, from the Weddell Sea to the Scotia Sea (*SI Appendix*, Fig. S6). The simulation was initialized with the hydrographic structure recorded by a CTD profile acquired in the deepest part of the southern Scotia Sea, and with zero initial flow. An inflow was specified at the Weddell Sea boundary, with hydrographic fields taken from our measurements along section A1, and a flow field into the domain computed from geostrophy. The simulation used a bathymetric data set for the region based on U.K., U.S. and German multi-beam measurements, with gaps filled by interpolating to GEBCO_2014 **(7)**, and the resulting data set interpolated to the model grid. Sponge boundary conditions were applied on all sides, adjusted to allow for mass balance within the domain. This simulation was run to quasi-steady state.

Second, a high-resolution (100 m in the horizontal, 20 m in the vertical) non-hydrostatic simulation was conducted in a smaller domain encompassing the main sill of the Orkney Passage (*SI Appendix*, Fig. S6). The simulation was initialized with the steady-state fields from the low-resolution simulation, interpolated to the high-resolution grid, and relaxed to those fields on all four boundaries. Laplacian viscosities of 5×10^{-2} m^2 s^{-1} in the vertical and 5×10^{-1} m^2 s^{-1} in the horizontal were adopted, with tracer diffusion implicit through the advection scheme. The simulation reached quasi-equilibrium after ~10 days, following which diagnostics were made.

Despite the many idealizations in the model configuration (steady-state side-boundary forcing, no surface fluxes, initialization from rest), the simulated fields display all the basic features of the boundary current revealed by the observations (*SI Appendix*, Fig. S7). These include: a dense cold layer flowing westward along the southern flank of the Orkney Deep, and northwestward over the main sill; a band of steep isopycnals and

weak stratification adjacent to the steep topography; and an area of flow reversal on the northeastern side of the main sill. This good agreement with the measurements applies to the distribution of potential vorticity too. Potential vorticity in the model is calculated as $q = (f\hat{\boldsymbol{k}} + \boldsymbol{\nabla} \times \boldsymbol{u}) \cdot \boldsymbol{\nabla} b$, without the need to adopt the approximations made in the observational analysis. As in the measurement-based diagnostics, q exhibits a reversal in sign adjacent to the topographic boundary (*SI Appendix*, Figs. S7a,c). A characterization of the overturning instabilities implied by this sign reversal **(8, 9)** confirms the view constructed from the observations (*SI Appendix*, Figs. S7b,d): a prevalence of conditions suitable for the development of gravitational and symmetric instabilities near topography, and a dominance of conditions favoring symmetric and centrifugal instabilities just above. The simulation's turbulent kinetic energy budget further supports the importance of symmetric and centrifugal instabilities in sustaining turbulent dissipation within the boundary current.

The pervasiveness of the sign reversal in potential vorticity at the onshore edge of the boundary current may be readily illustrated by considering the horizontal distribution of near-bottom q in the model (*SI Appendix*, Fig. S8a). Positive values of q extend along the entire Orkney Plateau slope contained within the high-resolution model domain and are accentuated near the main sill, suggesting a local topographic generation of the potential vorticity sign reversal. This suggestion is endorsed by diagnostics of the rate of frictional generation of q, defined as $\boldsymbol{\nabla} \cdot (\boldsymbol{F} \times \boldsymbol{\nabla} b)$, where $\boldsymbol{F} = \left(\nu_h \nabla_h^2 + \nu_z \frac{\partial^2}{\partial x^2}\right) \boldsymbol{u}$ is the frictional force on the ocean, and ν_h and ν_z respectively denote the frictional viscosities in the horizontal and vertical directions (*SI Appendix*, Fig. S8b). These reveal the widespread occurrence of large, generally positive rates of generation well in excess of 10^{-15} s^{-4} along the Orkney Plateau slope, with largest values in areas where the topography steepens and contracts horizontally. Further targeted simulations will

be pursued in a subsequent study to determine the precise conditions under which topographic generation of overturning instabilities at the boundary may unfold.

References

1. Haine TWN, Marshall J (1998) Gravitational, symmetric, and baroclinic instability of the ocean mixed layer. *J. Phys. Oceanogr.* **28**: 634-658. doi: 10.1175/1520-0485(1998)028<0634:GSABIO>2.0.CO;2.
2. Naveira Garabato AC *et al*. (2017) Vigorous lateral export of the meltwater outflow from beneath an Antarctic ice shelf. *Nature* **542**: 219-222. doi: 10.1038/nature20825.
3. Wenegrat JO, Callies J, Thomas LN (2018) Submesoscale baroclinic instability in the bottom boundary layer. *J. Phys. Oceanogr*. doi: 10.1175/jpo-d-17-0264.1.
4. Thomas LN (2008) Formation of intrathermocline eddies at ocean fronts by wind-driven destruction of potential vorticity. *Dyn. Atmos. Oceans* **45**: 252-273. doi: 10.1016/j.dynatmoce.2008.02.002.
5. Holmes RM, Thomas LN, Thompson L, Darr D (2014) Potential vorticity dynamics of tropical instability vortices. *J. Phys. Oceanogr.* **44**: 995-1011. doi: 10.1175/jpo-d-13-0157.1.
6. Marshall J, Adcroft A, Hill C, Perelman L, Heisey C (1997) A finite-volume, incompressible Navier-Stokes model for studies of the ocean on parallel computers, *J. Geophys. Res.* **102**: 5753-5766. doi: 10.1029/96jc02775.
7. Weatherall P. *et al*. (2015) A new digital bathymetric model of the world's oceans. *Earth Sp. Sci.* **2**: 331-345. doi: 10.1002/2015ea000107.
8. Thomas LN, Taylor JR, Ferrari R, Joyce TM (2013) Symmetric instability in the Gulf Stream. *Deep-Sea Res. II* **91**: 96-110. doi: 10.1016/j.dsr2.2013.02.025.

9. Hamlington PE, van Roekel LP, Fox-Kemper B, Julien K, Chini GP (2014) Langmuir - submesoscale interactions: Descriptive analysis of multiscale frontal spindown simulations. *J. Phys. Oceanogr.* **44**: 2249-2272. doi: 10.1175/jpo-d-13-0139.1.

Supplementary Figures

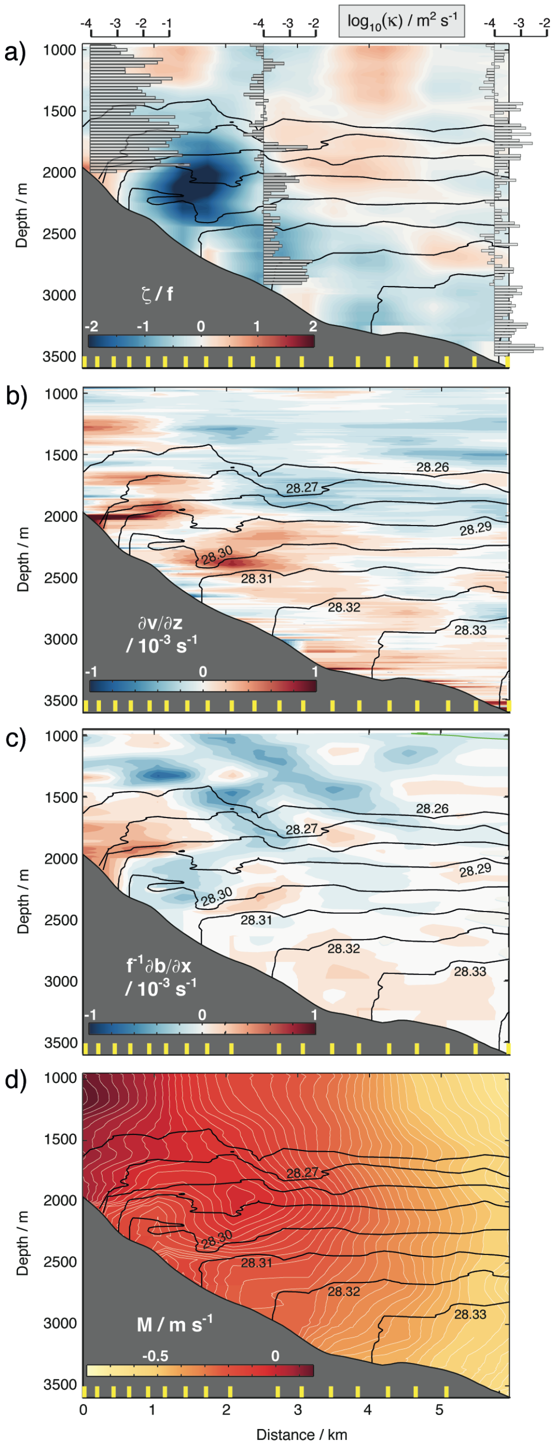

Figure S1 | Relative vorticity, vertical shear, lateral buoyancy gradient, and absolute momentum across the abyssal boundary current near the Orkney Passage sill. (a) Ratio of relative vorticity (ζ) to planetary vorticity (f) (color) and neutral density (black contours; only contours within Antarctic Bottom Water are shown) along section B3 (Figs. 1-2). The rate of turbulent diapycnal mixing (κ) is

indicated by shaded bars. The mean positions of measurement profiles are marked by yellow tick marks on the lower axis. (b) Vertical shear in the along-slope flow (v) (color) and neutral density (in kg m^{-3}, black contours). (c) Geostrophic vertical shear in the along-slope flow, $f^{1}\partial b/\partial x$, and neutral density (in kg m^{-3}, black contours). (d) Absolute momentum (M) (color) and neutral density (in kg m^{-3}, black contours).

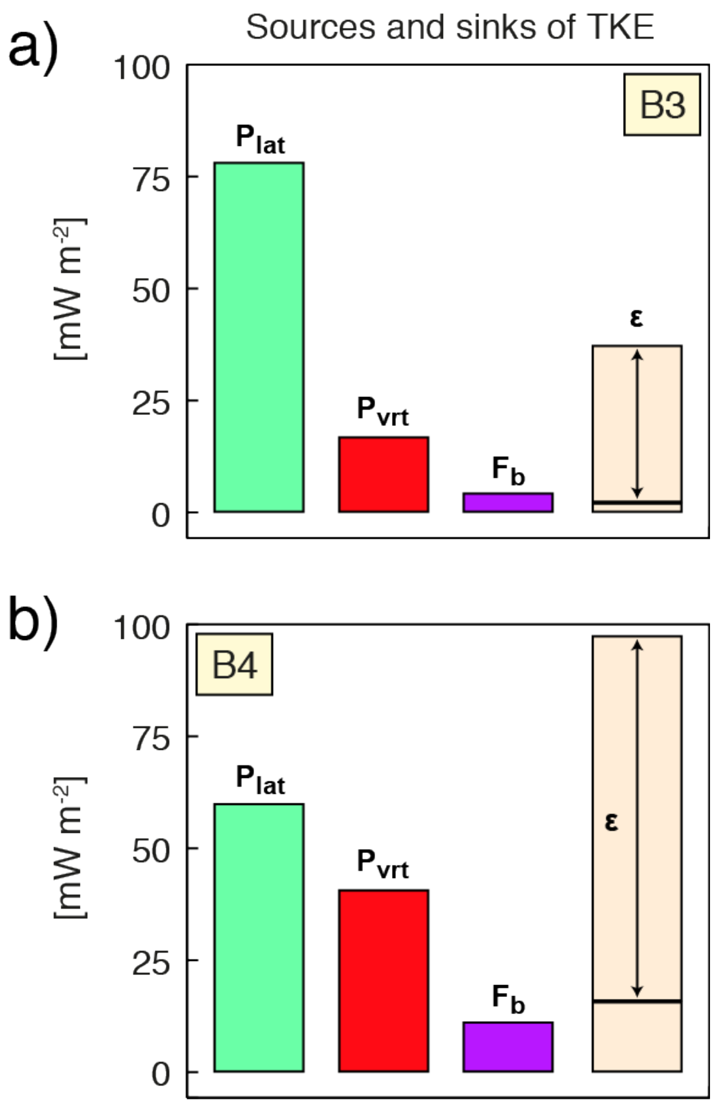

Figure S2 | Overview of sources and sinks of turbulent kinetic energy for the high-resolution transects near the Orkney Passage sill. Comparison between the vertically integrated (between depths of 1000 m, the upper bound of the CTD / LADCP measurements, and the ocean floor) rates of turbulent kinetic energy dissipation (ε) and of turbulent kinetic energy production associated with gravitational instability (F_b), symmetric instability (P_{vrt}) and centrifugal instability (P_{lat}) for (a) transect B3 and (b) transect B4. See *SI Appendix*, Supplementary Text.

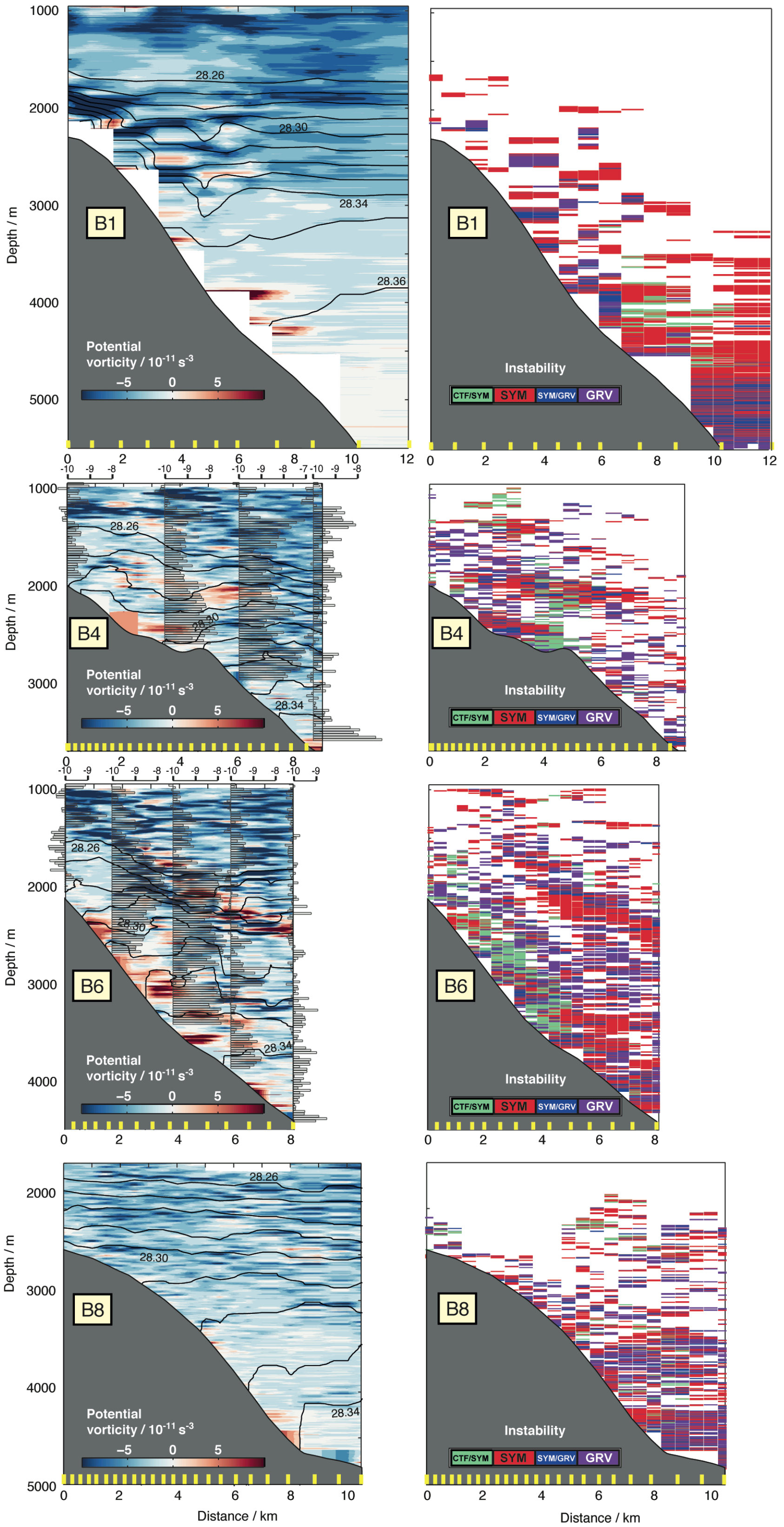

Figure S3 | Fine-resolution transects across the abyssal boundary current throughout the Orkney Passage region. Left column: potential vorticity (color) and neutral density (in kg m^{-3}, black contours; only contours within Antarctic Bottom Water

are shown) for sections (from top to bottom) B1, B4, B6 and B8 (Fig. 1). Profiles of the rate of turbulent kinetic energy dissipation, $\log_{10}(\varepsilon)$, are indicated by shaded bars for sections B4 and B6. Right column: instability type (CTF = centrifugal, SYM = symmetric, GRV = gravitational, and their hybrids; see *Materials and Methods*) diagnosed for sections (from top to bottom) B1, B4, B6 and B8. The mean positions of measurement profiles are marked by yellow tick marks on the lower axis of each panel.

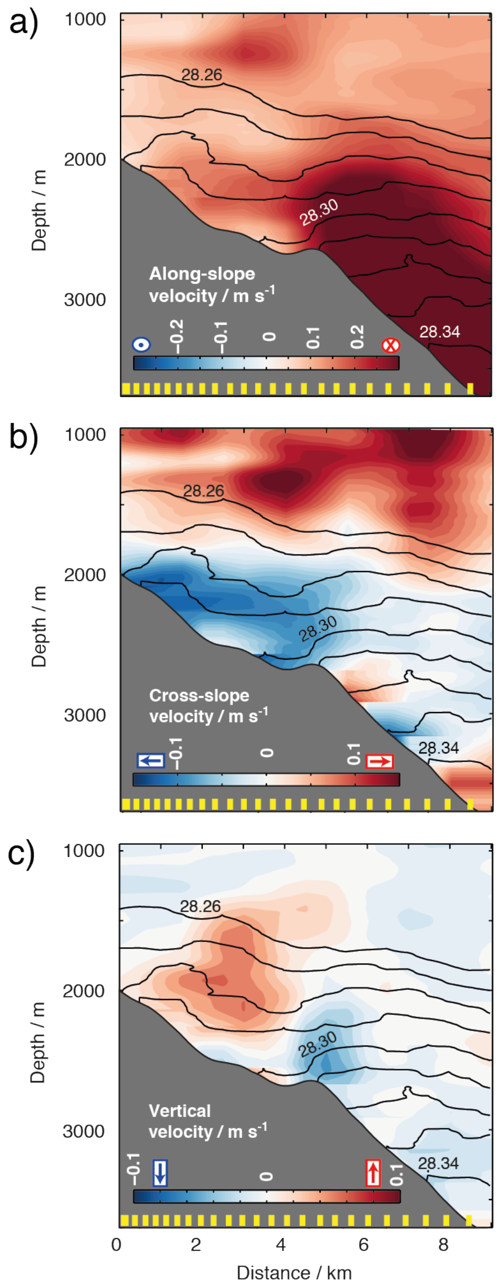

Figure S4 | Fine-resolution transect across the abyssal boundary current downstream of the Orkney Passage sill. (a) Along-slope velocity (color, with flow direction indicated above the color bar) and neutral density (in kg m^{-3}, black contours; only contours within Antarctic Bottom Water are shown) for section B4 (Fig. 3). The mean positions of measurement profiles are marked by yellow tick marks on the lower axis. (b) Cross-slope velocity (color) and neutral density (in kg m^{-3}, black contours). (c) Vertical velocity (color) and neutral density (in kg m^{-3}, black contours).

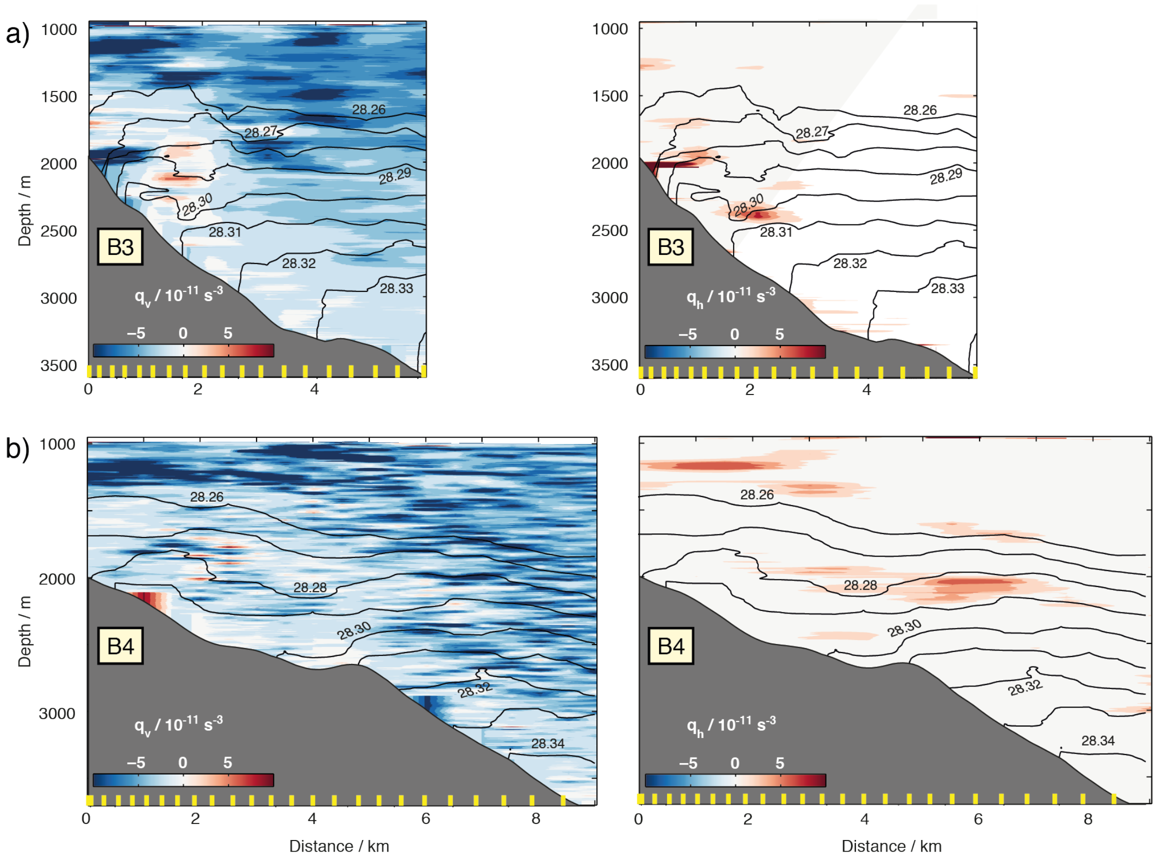

Figure S5 | Evolution of potential vorticity in the abyssal boundary current across the Orkney Passage sill. (a) Vertical (left) and baroclinic (right) contributions to the potential vorticity (in color) for section B3, upstream of the sill. (b) Vertical (left) and baroclinic (right) contributions to the potential vorticity (in color) for section B4, downstream of the sill. Neutral density, in kg m^{-3}, is shown by black contours in all panels (only contours within Antarctic Bottom Water are shown). The mean positions of measurement profiles are marked by yellow tick marks on the lower axis.

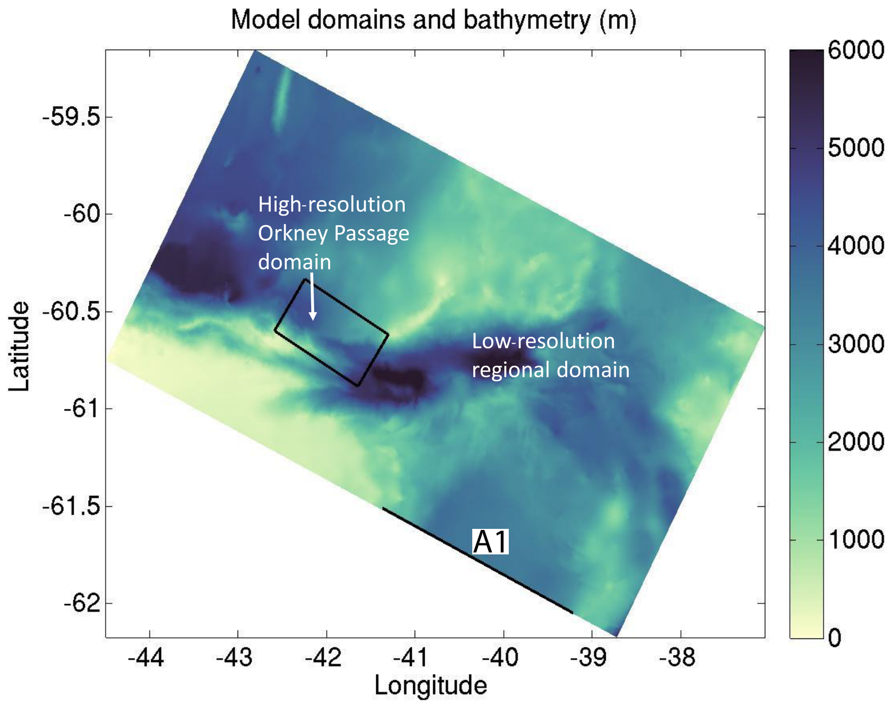

Figure S6 | Model configuration. A high-resolution Orkney Passage domain is nested within a low-resolution regional domain. The section determining the inflow boundary condition is marked and labeled (A1).

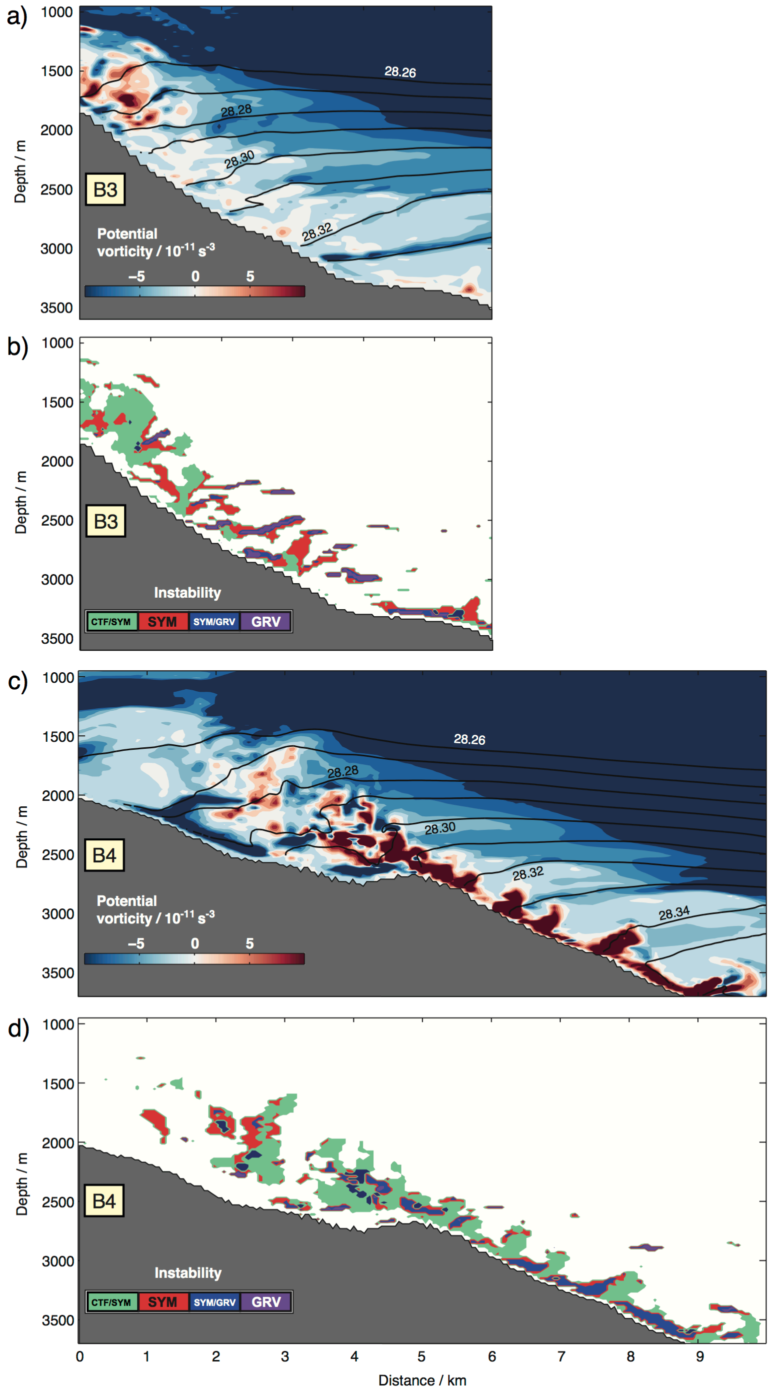

Figure S7 | Modeled potential vorticity and overturning instabilities near the Orkney Passage main sill. Snapshot of potential vorticity (color) and neutral density (in kg m^{-3}, black contours; only contours within Antarctic Bottom Water are shown) along the position of (a) the B3 transect and (c) the B4 transect at 10 days after the spin-up of the high-resolution simulation. Corresponding instability type (CTF = centrifugal, SYM = symmetric, GRV = gravitational; see *Materials and Methods*) diagnosed along the position of (b) the B3 transect and (d) the B4 transect for the same snapshot.

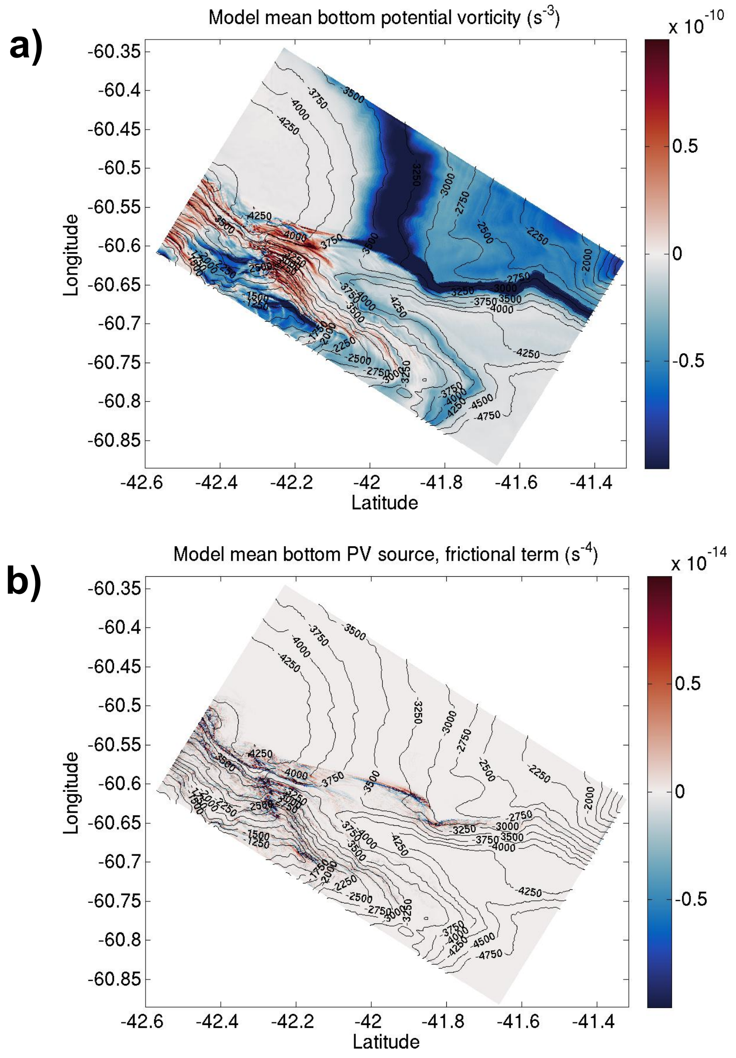

Figure S8 | Modeled topographic generation of potential vorticity near the Orkney Passage main sill. (a) Snapshot of potential vorticity averaged over the deepest 100 m (color) at 10 days after the spin-up of the high-resolution simulation. (b) Rate of frictional generation of potential vorticity (color) at the ocean floor for the same snapshot. Isobaths (in m) are indicated by contours in both panels.

Supplementary Movie

Movie S1. A virtual fly-through of the second of ALR's two missions on the slope of Orkney Passage main sill (mission 44, 18 - 22 April 2017). Bathymetry is shown by blue shading. The red line indicates the track followed by ALR. Black vectors show horizontal velocity averaged in the range 125-150 m above the ocean floor. Purple circles denote the neutral density measured by the ALR at ~90 m above the ocean floor. Each velocity and density value represents an average of measurements over 0.5 km of ALR track.
